# Some Modern Continental Hospitals

**Published:** 1908-08-15

**Authors:** 


### HOSPITAL ADMINISTRATION.

### CONSTRUCTION AND ECONOMICS.

### SOME MODERN CONTINENTAL HOSPITALS.

#### II.—THE RUDOLPH VIRCHOW HOSPITAL AT BERLIN.

Operating Theatres.

The operating theatres at the Virchow are situate in a large block placed in the centre of the surgical pavilions and connected by long corridors, on the one side with the surgical division for women, and on the other with the surgical division for men. These joining corridors have given rise to some discussion, and at first sight it seems difficult to imagine an operating theatre standing alone amidst a system of pavilions and totally separated from them. Yet that is the case at the Eppendorf Hospital, Hamburg, and it appears to be an ideal which should be realised wherever possible. Such long corridors are badly ventilated and have attached to them numerous disadvantages which the single argument in their favour—namely, that they are necessary for the carrying of patients to and from an operation—hardly outweighs. It is a remarkable fact, as Kummel has shown, that the statistics in regard to post-operative broncho-pneumonias are more favourable (2.5 per cent.) at the Eppendorf Hospital, where patients have to be carried through the open air to and from the unconnected central operating theatre, than in hospitals where such open-air transport is obviated by closed corridors, or where the theatre is itself placed alongside, or even in, a ward block. The necessary covering, for use in wet weather, can be easily provided by a tented carriage or by the use of covered corridors entirely open at the sides—the type of verandah corridors which exists at some Italian and Spanish hospitals. At the Virchow the connecting corridors are not efficiently ventilated and prevent full and perfect aëration of the central block to which they are attached.

This central block consists of two large operating theatres and two smaller rooms, attached to which are a number of other rooms used for sterilising, for bandaging, for plaster work, and for the staff, while above these smaller rooms are the rooms for the attendant. As in the central block of the pavilions, there are too many single rooms, and the visitor gets the impression that half of them, at present at least, are superfluous. The main theatres correspond closely. They are large, lofty, airy rooms, open to the air on the north side by large alcove ground-glass windows, which admit light from the east and west. These windows reach from floor to ceiling, and even on a murky day give admirable, diffused light, and can be freely opened to permit of very excellent ventilation by natural means. This natural ventilation has hitherto been found to be quite sufficient, and the artificial methods, which are there if required, have not been used. For work at night or on cloudy days, head lights and side lights are used. These are all placed without the room in ground-glass chambers. The roof of the theatre, in fact, is a ground-glass compartment in which are placed several strong, "central-reflecting" electric lights, while similar lights, at a slightly lower level, are arranged along the sides in the wall. No lamp is directly in the room, and the accumulation of dust on the reflectors, etc., is thus efficiently prevented. The advantage of this method is very great. The light is admirably diffused throughout the room, and the lamps are so arranged that shadows are reduced to a minimum, while the operator and those working in the room are not inconvenienced by glare or heat. As arranged, the Virchow operating theatre is probably the best lit theatre to be seen anywhere, and the arrangements have been found to work admirably.

Recognising that it is virtually impossible to obtain, under existing conditions in a large city, an atmosphere entirely free from dust and at the same time to have adequate ventilation, the designers have not attempted to strive for the unrealisable. One misses, therefore, the finnicky superfluities which are to be found in some modern operating rooms—the aseptic air screens and filters, the glass coverings for everything, and the inlets for "sterile air." The warming is by means of hot water and steam pipes, a double system, and the radiators are open and not enclosed in glass. The walls are of cement, tiled half-way and painted a dull grey; the radiators are painted a military grey colour; the floor is tiled. Edges and joints are, as far as possible, avoided, and, as in all modern theatres, the fittings are so arranged that the room can be easily cleaned by means of the hose. The instruments—in charge of a special "instrument sister" who attends all operations and hands out the sutures and instruments—are kept in glass cases in a recess out of the main room. Several hand-basins, the water of which is laid on in pipes which are controlled by hand as well as foot pressure, are ranged along the south wall. The drain pipes are large and covered with movable gratings. In the centre of the room is the operating table, of simple yet admirable construction, working on ball bearings, so that it can be raised or lowered by foot pressure to any desired position by the operator himself. Hot-water circulators are not used, for the temperature of the room can be regulated to a nicety by means of the radiators. On the west wall are the pipes for the supply of antiseptics, sterile saline solution, distilled water (both hot and cold), the reservoirs for all these being in the adjoining room.

From the operation theatre one enters, through a door in the west wall into the smaller operating room. Next to it is the sterilising room, with several sterilisers—one for instruments, another for bandages, another for normal saline and distilled water. Dressings are sterilised here and brought into the main theatre by the sister in special canisters. In no case do they come from the wards. Here, too, is kept a Brauer's high-pressure cabinet for operations on the lungs, but I find that it has not as yet been used. Through a door in the south wall one enters the anæsthetising room, where the special anæsthetising apparatus, both for lumbar and general anæsthesia (the latter being German modifications of the Vernon-Harcourt type) are kept. Next to it is the preparation room, and opening into it the splint and bandage room. Rooms for the staff, a retiring room for the surgeon, an overall and shoe room (rubber shoes are invariably worn at operations here), and an emergency *x*-ray room are close at hand. The lavatory accommodation is provided for at the back of the theatre, behind the corridors. The main features of the operating rooms at the Virchow Hospital are their simplicity and their effectiveness. The maximum result appears to have been attained with the minimum of elaboration and complexity. Yet all this apparent simplicity is really the outcome of careful thought, much experiment, and accurate comparison of various methods. For that reason the rooms are interesting, as they probably represent the best type of operating theatre that it is possible to construct under existing conditions. The faults, if they are faults, are to be found, not in the theatres themselves, but in the block to which they are attached, and consist, as we have already stated, in the corridors and in the number of single rooms. The theatres themselves are in every way admirable, both from the point of view of the surgeon and from the point of view of the architect. There is a large hydrotherapeutic department very fully equipped.

### The Hospital Staff.

The staff consists of a general director, the *Verwaltungs Director*, a medical director in charge of the medical side, and a surgical director in charge of the surgical side; three chief physicians and surgeons, and thirty-six assistants who have rooms, usually comprising a bed and sitting room and in some cases a special study. There are a matron (a separate flat in the Nurses' Home), 24 ward sisters (each a bed-sitting-room), 130 nurses (in two-bedded rooms), 40 probationers (in six-bedded dormitories), 2 midwives (each a separate room). There is one head, three assistant, and three supernumerary dispensers, who have rooms near the dispensary. The theatre assistant, in charge of the operating rooms, has a flat—consisting of bed-sitting-room, spare room, and kitchen—over the operating rooms. The post-mortem-room attendant has a similar flat in the pathological building. The "chief secretary," who is on day duty, has a special flat in the administrative block; the two "night secretaries" have rooms in the same block. The chief engineer and the chief machine inspector have flats close to the administrative block. The works inspector, overseer, and head cook have each a separate house. The plumber, head fitter, two head stokers, and the head gardener have each a special room. In the kitchen block reside two assistant cooks (each a room), and 26 under-cooks (in a dormitory). In each of the pavilions reside two male ward attendants. In addition, there are one head disinfecting-room attendant, who has a separate flat, one head warder, and 27 assistant warders (who have each a separate room), 18 ward attendants, 27 ward maids, and 36 housemaids (who occupy large dormitories), two porters, each residing in a separate flat attached to his respective lodge, and a messenger who has a room in the administrative block. The *personnel* of the whole institution—exclusive of day labourers such as assistants to the gardeners, carpenters, plumber and fitters—thus numbers between 400 and 500 persons.

### Finances and Statistics.

The latest available return of the finances of the hospital and the administrative report are contained in the *Verwaltungsbericht für das Etatsjahr*, 1906 (No. 18), courteously placed at my disposal by the directorate. From this report I take the following interesting particulars: Number of beds in occupation, 975; number of patients treated to end of March 1907 (it must be remembered that the institution was only opened in October 1906 and that these figures are therefore merely a half-yearly return, thus the number of occupied beds is at present 1,600), males, 2,758; females, 1,184; total deaths, 239; discharged, 2,926; largest monthly return of patients admitted, 789 (March); average daily admissions, 21; average of daily number of occupied beds, 589; average length of patients' stay in hospital 25 days. Financial: revenue from various sources (irrespective of rates), 5,536 marks, or approximately £277, made up of sale of refuse, straw, water, etc.; expenditure, administrative (including personal and administrative costs), £3,000; wages, £2,500; salaries, £3,500; medicines and extras, £1,500; "spiritual care and instruction of patients," £25; food, clothing, laundry, and bed linen of patients, £9,300; housekeeping expenses (heating, lighting, water, repairs, etc.), £10,500; gratuities paid to patients on discharge (poor-box fees), £60; costs of burial, £13; total expenditure, including small sums not enumerated above, £32,200, working out at cost per patient per day 6s. (as against the average of 3s. for the other Berlin hospitals). All these figures, it must be borne in mind, are approximate only.

Return No. 3. of the same report gives more detailed information regarding the expenses. Leaving the administrative expenses and taking only those given under the heading "treatment of patients," one finds the following figures regarding the salaries of the staff: Directors each 6,000m.; chief physician in the gynæcological department, 2,500m.; ditto isolation, 2,000m.; bacteriologist, 1,500m.; chemist, 1,500m.; *x*-ray operator, 2,000m.; chief assistants in the genito-urinary department, 2,000m.; pathologist, 5,000m.; assistant physicians (with extras), average 1,200m. each; and dispenser, 1,200m. The wages of the attendants came to 82,400m. (187 persons); 2,145m. were expended in Christmas presents to the attendants. The cost of food works out at the following interesting figures per head per day: For the staff, 2.65m.; for the attendants, 1.20m.; for the patients, .95m.—figures which correspond very closely with those given for the Moabite, Friedrichshain, and Urban hospitals.

### Some General Conclusions.

As an example of a modern up-to-date institution, the Rudolph Virchow Hospital is not ideal, and it fails because it is on too large and gigantic a scale to be adequately supervised and efficiently managed. As a hospital it fails to realise the ideal of hospital construction for the same reason, and it inevitably suffers when compared with other modern institutions such as the great infirmaries at Hamburg and the newer suburban hospitals in Germany and elsewhere. Its immensity necessitates an unduly large staff, an unusual amount of care and attention, and an excessive expenditure and outlay upon repairs and alterations. As a hospital it is a striking lesson in favour of smaller and less gigantic and less palatial institutions. Its very largeness has made its pavilions single hospitals, which are not the best examples of their kind, and no one who traverses its wide pathways and admires its beautiful proportions can fail to regret that so much time, money, and attention have been expended upon the building and outfitting of an institution which is by no means ideal. Whether it will become a white elephant for the Municipality of Berlin remains to be seen. With its growing population and with the risk of an epidemic recurring now and again, the city has need of adequate accommodation for patients, and such accommodation it has certainly obtained through the building of this institution. Whether or not the need would have been provided for as adequately and far less extravagantly by the building of a series of smaller hospitals; whether or not it is in principle bad to mass the metropolitan sick, especially in times of epidemics, in one huge establishment such as this; and finally whether or not the cost of keeping in repair and in order these numerous beds waiting for possible epidemic patients is one which the ratepayers will regard with equanimity in years to come, are questions which only time can settle. As it stands, the Rudolph Virchow Hospital is an old experiment repeated in a new manner and on new lines, and those interested in hospitals will look forward to seeing whether it proves a success or a failure.

